# UCP1-independent glucose-lowering effect of leptin in type 1 diabetes: only in conditions of hypoleptinemia

**DOI:** 10.1152/ajpendo.00253.2019

**Published:** 2019-11-19

**Authors:** Petr Zouhar, Günaj Rakipovski, Muhammad Hamza Bokhari, Oliver Busby, Johan F. Paulsson, Kilian W. Conde-Frieboes, Johannes J. Fels, Kirsten Raun, Birgitte Andersen, Barbara Cannon, Jan Nedergaard

**Affiliations:** ^1^Department of Molecular Biosciences, The Wenner-Gren Institute, Stockholm University, Stockholm, Sweden; ^2^Department of Adipose Tissue Biology, Institute of Physiology CAS, Prague, the Czech Republic; ^3^Global Drug Discovery, Novo Nordisk A/S, Måløv, Denmark

**Keywords:** glucagon, insulin receptor antagonist, leptin, thermoneutrality, type 1 diabetes, uncoupling protein 1

## Abstract

The possibility to use leptin therapeutically for lowering glucose levels in patients with type 1 diabetes has attracted interest. However, earlier animal models of type 1 diabetes are severely catabolic with very low endogenous leptin levels, unlike most patients with diabetes. Here, we aim to test glucose-lowering effects of leptin in novel, more human-like murine models. We examined the glucose-lowering potential of leptin in diabetic models of two types: streptozotocin-treated mice and mice treated with the insulin receptor antagonist S961. To prevent hypoleptinemia, we used combinations of thermoneutral temperature and high-fat feeding. Leptin fully normalized hyperglycemia in standard chow-fed streptozotocin-treated diabetic mice. However, more humanized physiological conditions (high-fat diets or thermoneutral temperatures) that increased adiposity — and thus also leptin levels — in the diabetic mice abrogated the effects of leptin, i.e., the mice developed leptin resistance also in this respect. The glucose-lowering effect of leptin was not dependent on the presence of the uncoupling protein-1 and was not associated with alterations in plasma insulin, insulin-like growth factor 1, food intake or corticosterone but fully correlated with decreased plasma glucagon levels and gluconeogenesis. An important implication of these observations is that the therapeutic potential of leptin as an additional treatment in patients with type 1 diabetes is probably limited. This is because such patients are treated with insulin and do not display low leptin levels. Thus, the potential for a glucose-lowering effect of leptin would already have been attained with standard insulin therapy, and further effects on blood glucose level through additional leptin cannot be anticipated.

## INTRODUCTION

Leptin treatment ameliorates hyperglycemia in rodents with insulin deficiency, an effect that is independent of food intake and glucose loss in urine. Accordingly, the use of leptin as a treatment for type 1 diabetes has been suggested (46).

To explain the blood glucose-lowering effect, several mechanisms, both peripherally and centrally mediated, have been proposed by several groups (reviewed in Ref. 7). In principle, leptin may lower blood glucose directly or indirectly by inhibiting hepatic glucose production and/or by induction of glucose uptake, storage, and combustion in certain tissues, including the major thermogenic organ: brown adipose tissue (BAT). Elucidation of the mediation of the glucose-lowering effect of leptin is clearly of both theoretical and therapeutic interest. For this, diabetic models may be used.

The standard streptozotocin-induced rodent model of type 1 diabetes presents, however, with some issues. One is that the model leads to a principally total loss of insulin production, unlike the successive development seen in humans. Another is that the animals rapidly lose fat mass and therefore become hypoleptinemic. In contrast, as insulin is a strong inducer of leptin secretion (reviewed in Ref. 43), and as patients with type 1 diabetes are treated with insulin, diabetic patients without diabetic ketoacidosis usually have normal leptin levels (21–23). To harmonize the rodent and human data and to examine the efficiency of leptin treatment in a metabolic state more closely resembling the majority of patients with type 1 diabetes, less catabolic rodent models of type 1 diabetes that also display higher leptin levels are needed.

In the present investigation, such models were obtained either by a combination of a less harsh streptozotocin treatment and high-fat feeding at thermoneutrality or by a combination of treatment with the insulin receptor antagonist S961, which functionally mimics an absence of insulin (41), and high-fat feeding. Our results suggest that the glucose-lowering effects of leptin are attenuated in mice with normal or elevated leptin levels and that the glucose-lowering effect of leptin is associated with, and probably caused by, effects of leptin on glucagon levels. In a broader perspective, the present results weaken the prospects of using leptin in the routine treatment of type 1 diabetes.

## RESEARCH DESIGN AND METHODS

### Animals

The layout of the project is based on investigations of leptin effects in a series of different conditions, referred to as *studies 1*–*6*. *Studies 1*–*4* were conducted in the animal facility of Stockholm University (Sweden) on single-caged animals, whereas *studies 5* and *6* were performed in the Novo Nordisk animal facility in Måløv (Denmark) and the animals were group housed (3–4 per cage). All animals were males housed in a 12/12-h light-dark cycle (light starting at 7:00 AM) with ad libitum access to food and water. All experimental procedures were approved by the regional Ethics Committee for North Stockholm or the internal ethics committee of Novo Nordisk A/S.

In *studies 1*–*3*, C57Bl/6J animals (Scanbur, *studies 1* and *2*) and uncoupling protein-1 (UCP1)^+/+^ and UCP1^−/−^ (own breeding at Stockholm University, C57Bl6N background, *study 3*) were fed a standard chow diet R70 (Lactamin AB) and acclimated to 30°C for 5–7 wk (starting at 11–13 wk of age). In *study 4*, mice were fed a high-fat diet D12451 (Research Diets, 45% energy as fat) and acclimated to either 21°C or 30°C for 20 wk (starting at 10 wk of age). In *study 5*, C57Bl/6J mice (Taconic, 8–10 wk old at arrival) were housed at 21°C and fed chow diet (Altromin no. 1324). In *study 6*, dietary obese C57Bl6J mice (Taconic, 27 wk old at arrival) fed high-fat diet D12492 (Research Diets, 60% energy as fat) were housed at 21°C.

### Induction and Treatment of Hyperglycemia

Chow-fed animals (*studies 1*–*3*, at age 16–18 wk) were injected with low doses of streptozotocin (50 mg/kg) once daily intraperitoneally for 5 days. In the high-fat-fed mice (*study 4*), hyperglycemia was induced at an age of 28–30 wk by similar low doses of streptozotocin (3 × 60 mg/kg body weight).

In separate sets of experiments, Alzet osmotic mini-pumps filled either with vehicle or with insulin receptor antagonist S961 (0.35 nmol/μL, 0.25 μL/h) were implanted subcutaneously either in chow-fed (*study 5*) or in high-fat-fed (*study 6*) mice at the age of 11 wk or 28–30 wk, respectively.

After development of hyperglycemia (2–3 wk after streptozotocin or 4 days after S961-containing mini-pump implantation), mice were treated once daily subcutaneously either with vehicle or with long-acting acylated human leptin [1 mg/kg if not otherwise stated; developed by Novo Nordisk A/S (27)] for 1 (*studies 2*, *3*, *5*, *6*) or 3 wk (*studies 1* and *4*). In vitro, the long-acting leptin analog is equipotent and has the same binding affinity to the leptin receptor as wild-type human leptin: t_1/2_ = 3–4 h in mice (27).

### In Vivo Measurements

During the entire time of the experiment, tail blood glucose levels were measured using glucometers (AccuChek Aviva; values out of range were remeasured by Nova StatStrip Xpress glucometer) in *studies 1*–*4* or using Biosen system solution (EKF Diagnostics) in *studies 5* and *6*. Blood glucose was always measured in the nonfasted state in the morning before the daily administration of the compounds. Body weight was obtained daily, and food intake was measured once to twice weekly as indicated in specific figures (only in *studies 1*–*4*, in which the mice were single caged). In studies with 3-wk leptin treatment (*studies 1* and *4*), 10 μL tail blood samples were collected in 500 μL hemolyzing reagent (Roche/Hitachi) 3 times during the experiment (before streptozotocin, before leptin/vehicle dosing, and after dosing) and were used for determination of glycated hemoglobin A1c (HbA1c) using Tina-quant Hemoglobin A1c Gen. 3 kit (Hoffmann-La Roche) and Cobas 6000 analyzer (Roche Diagnostics). In *studies 1*–*4*, body composition was measured with an EchoMRI analyzer before streptozotocin and before and after leptin/vehicle dosing.

In *study 2*, a pyruvate tolerance test was performed on *day 8* of treatment. Mice were fasted for 15 h and then injected intraperitoneally with pyruvate (1.89 g/kg of lean body mass, corresponding approximately to 1.5 g/kg body weight). Blood glucose was measured just before pyruvate injection and 15, 30, 60, and 120 min after.

In *study 3* (comparing UCP1^+/+^ and UCP1^−/−^ mice), oxygen consumption was recorded every 2 min using an indirect calorimetry system (Somedic). Mice were kept in insulated chambers at 30°C for 24 h (starting at 1:00 PM) on the same light-dark cycle as in the housing room. Three hours before the end of the measurements, at 11:00 AM, nonshivering thermogenesis was maximally stimulated by intraperitoneal injection of the β_3_-adrenergic agonist CL316,243 (1 mg/kg). Control groups of UCP1^+/+^ and UCP1^−/−^ mice untreated with streptozotocin were measured in parallel.

In *studies 5* and *6*, blood samples were collected in the morning without anesthesia from the facial vein before the start of the treatment for analysis of plasma hormone levels. At the end of the experiment, these animals were anesthetized with isoflurane, and a terminal blood sample was collected from the sinus orbital vein.

In *studies 1*–*4*, animals were finally anesthetized with isoflurane (between 9:00 and 11:00 AM, ~24 h after last leptin or vehicle injection) and then decapitated, and blood was collected by carotid bleeding. Tissues were collected for quantitative PCR (qPCR) and Western blot analyses.

### Plasma Hormone Levels

Blood was collected as described above and mixed with EDTA (to a final concentration of approx. 1.2 mg/mL) and cOmplete Mini protease inhibitor mix (Sigma Aldrich). Samples were centrifuged 10 min 3,000 × *g* at 4°C and plasma was separated.

Levels of the following hormones were measured in the plasma samples: insulin (Ultrasensitive insulin ELISA assay, Crystal Chem Inc.), leptin [Leptin (mouse) AlphaLISA Detection Kit, Perkin Elmer, which does not recognize the leptin analog used for the treatment], corticosterone (Corticosterone ELISA kit, DRG International), and IGF1 and glucagon [in-house-developed luminescence oxygen channeling immunoassays (LOCI)].

The LOCI assays were performed according to the following protocol: plasma samples (2 μL plasma for glucagon or 5 μL plasma for IGF1 pretreated with a proprietary “releasing agent” to displace all IGF1-binding proteins) were applied to 384-well LOCI plates. A mixture (15 μL) of biotinylated antibodies (mAb GLU 2F7 against glucagon or mAb 1F6 against IGF1) and acceptor beads conjugated with another antibody (mAb GLU 1F120 against glucagon or mAb 4F38 against IGF1) were added to each well. After 1-h incubation, 30 μL streptavidin-coated donor beads (67 μg/mL) were added. During the following 30-min incubation, a bead-aggregate-immune complex was formed in the presence of the analyte (i.e., glucagon or IGF1), placing the donor and acceptor beads in immediate proximity. Using an EnVision plate reader with a bandwidth of 520–645 nm, samples were illuminated with a 680 nm laser, resulting in the formation of singlet oxygen on the donor bead. In the presence of the analyte, the singlet oxygen is channeled to an adjacent acceptor bead, triggering chemiluminescence. The amount of light generated is proportional to the concentration of the analyte. All steps were carried out at standard room temperature (21–22°C). The lower limit of quantification of glucagon is 4 pM. The dynamic range of the IGF1 assay is 20–2,000 ng/mL.

If the measured plasma hormone levels were below detection limits, the detection limit was used for calculations.

### Quantitative RT PCR

Gene expression was analyzed in samples from *study 1*. It was carried out separately for brown and white adipose tissue (BAT/WAT) and for liver, using two different protocols. In BAT and inguinal WAT, qPCR was performed as described before (13). Data were normalized to general transcription factor IIB (TFIIB) as a housekeeping gene. For primer sequences, see Table 1.

**Table 1.** qPCR primer sequences used for analysis of gene expression in BAT and inguinal WAT

Gene	Gene ID	Fw Sequence	Rv Sequence
TFIIB	229906	TGGAGATTTGTCCACCATGA	GAATTGCCAAACTCATCAAAACT
Ucp1	22227	GGCCTCTACGACTCAGTCCA	TAAGCCGGCTGAGATCTTGT
PGC1α	19017	GAAAGGGCCAAACAGAGAGA	GTAAATCACACGGCGCTCTT
Dio2	13371	CTGCGCTGTGTCTGGAAC	GGAATTGGGAGCATCTTCAC
Cidea	12683	GCCTGCAGGAACTTATCAGC	AGAACTCCTCTGTGTCCACCA

BAT, brown adipose tissue; Cidea, cell-death-inducing DNA fragmentation factor α subunit-like effector A; Dio2, type 2 iodothyronine deiodinase (“deiodinase 2”); Fw, forward; PGC1α, peroxisome proliferator-activated receptor γ-coactivator 1α; qPCR, quantitative PCR; Rv, reverse; TFIIB, general transcription factor IIB; Ucp1, uncoupling protein 1; WAT, white adipose tissue.

RNA from liver was isolated using RNeasy Mini kit (Qiagen) and transcribed to cDNA using an iScript cDNA Synthesis Kit (Bio-Rad). qPCR was then performed using a light cycler Viia7 and TaqMan system (Fisher Scientific), including TaqMan Fast Advanced Master mix and specific primers with catalog numbers listed in Table 2. Data were normalized to β actin as a housekeeping gene.

**Table 2.** qPCR primers used for analysis of gene expression in liver

Abbreviation	Gene	Gene ID	ThermoFisher Assay ID
Actin b	β-actin	11461	Mm02619580_g1
PEPCK	Phosphoenolpyruvate carboxykinase 1	18534	Mm01247058_m1
Glut2	Glucose transporter 2	20526	Mm00446229_m1
GK	Glycerol kinase	14933	Mm00433896_m1
Fasn	Fatty acid synthase	14104	Mm00662319_m1
SCD1	Stearoyl-coenzyme A desaturase	20249	Mm00772290_m1
FoxA2	Forkhead box protein A2	15376	Mm01976556_s1
FGF21	Fibroblast growth factor 21	56636	Mm00840165_g1
LepR	Leptin receptor	16847	Mm00440181_m1
IGF1	Insulin-like growth factor 1	16000	Mm00439560_m1
IGFBP2	IGF1-binding protein 2	16008	Mm00492632_m1

### Western Blot

In *study 1*, proteins from one entire depot of BAT or inguinal WAT were isolated and quantified as described previously (13). Also, SDS-PAGE, electrophoretic transfer, blocking, probing with respective antibodies, and visualization of the staining were performed according to the same protocol. Four μg of BAT protein or 9 μg of inguinal WAT protein were loaded on the gel. To check for equal protein loading, Ponceau Red staining was applied immediately after the semidry transfer. After quantification of band intensity, values were normalized to a BAT standard on each gel and then multiplied by the amount of protein in the entire tissue, to obtain total UCP1 in the tissue, reflecting maximal thermogenic capacity. An in-house developed primary UCP1 antibody (rabbit polyclonal, raised against the C-terminal decapeptide, 1:20,000) and secondary antirabbit horseradish peroxidase-linked IgG antibody (Cell Signaling, 1:20,000) were used.

### Statistics

Statistical analysis was performed using Graph Pad Prism. Data are represented as arithmetic means ± standard error (in column graphs, individual data points are also shown). The statistical methods used are indicated in figure legends. *, **, ***, and **** indicate *P* ≤ 0.05, *P* ≤ 0.01, *P* ≤ 0.001, and *P* ≤ 0.0001, respectively.

## RESULTS

### The Streptozotocin Model of Type 1 Diabetes Examined at Thermoneutrality

Earlier studies of the glucose-lowering effects of leptin have mostly been performed at standard room temperature (approximately 20°C–22°C). Although this temperature is thermoneutral for fully clothed humans (i.e., humans who have minimal additional energy demands for thermogenesis to defend body temperature), it imposes significant cold stress to small laboratory rodents (11, 14), increasing their energy cost for maintaining a stable body temperature and through this possibly accelerating body weight loss after induction of insulin deficiency. Therefore, in the present experiment, mice were housed at thermoneutrality (i.e., ∼30°C for a normal mouse) to better mimic the metabolic situation in humans (11).

In streptozotocin-induced models of diabetes, the diabetic state is often induced by injection of a single high dose of streptozotocin (6). However, we anticipated that employing multiple low doses of streptozotocin could create a less catabolic model of insulin deficiency that better represents hormonal levels of human patients on insulin therapy, with progressive β cell loss and prolonged survival (6). We therefore used a protocol with repeated injections of low doses of streptozotocin over the course of 5 days (various methods of streptozotocin treatment are reviewed in Ref. 6).

**Fig. 1.**
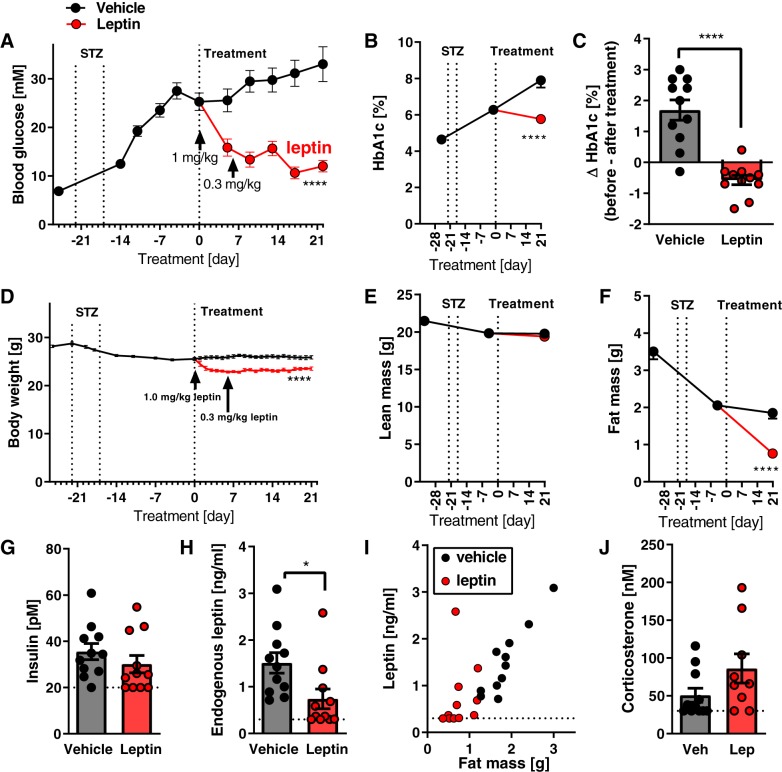
Effects of leptin on lean mice with streptozotocin-induced diabetes at thermoneutrality. Time course of nonfasted morning blood glucose after streptozotocin (STZ) exposure and during leptin treatment [arrows indicate start of leptin (lep)/vehicle (veh) treatment and lowering of leptin dose] (*A*). Time course of glycated hemoglobin A1c (HbA1c) levels (*B*). Changes in HbA1c during leptin treatment (between *days 0* and *21*) (*C*); here and in similar graphs, individual values and means ± SE are indicated. Time course of changes in body weight (*D*), lean body mass (*E*), and fat mass (*F*). Body weight at *day 0* was significantly lower than at *day −21* (*t* test; *P* < 0.001). Nonfasting plasma levels of insulin at the time of dissection (morning) (*G*); dotted horizontal line in this and similar graphs represents detection limit of the assay. Nonfasting plasma levels of endogenous leptin at the time of dissection (*H*); the leptin analog used for injections is not recognized by the assay. Correlation between fat mass and endogenous leptin levels (*I*). Nonfasting plasma levels of corticosterone (*J*). *Study 1*; *n* = 10–11. Statistics: Student’s *t* test (*C*, *G*, *H*, and *J*); repeated measures two-way ANOVA alone (*A* and *D*; the indicated significance reflects all measurements starting at *time 0* taken together), or together with Sidak’s multiple comparison test for comparison of individual time points (*B*, *E*, and *F*). * and ****Significant effects of leptin treatment (*P* < 0.05 and < 0.0001, respectively).

The streptozotocin treatment resulted in a gradual development of hyperglycemia and a rise in HbA1c (Fig. 1, *A* and *B*, curve until *day 0*). However, despite minimal energy demands for thermogenesis, the animals still lost some body weight (Fig. 1*D*); the fat mass especially was decreased (Fig. 1, *E* and *F*).

At the end of the experiment (i.e., 6 wk after the start of the streptozotocin treatment), there was still a low, but generally detectable, amount of insulin (20–60 pM) (Fig. 1*G*, *left*). The endogenous leptin level was also relatively low (Fig. 1*H*, *left*) and correlated closely with fat mass (Fig. 1*E*, closed points). In contrast with what has been observed in more catabolic models (38), corticosterone was in the normal range in this model (Fig. 1*J*).

Thus, multiple low doses of streptozotocin at thermoneutrality resulted in diabetic mice with long survival. This protocol could therefore be considered a reasonably improved model of the human type 1 diabetic situation.

### Antidiabetic Effects of Leptin in Hypoleptinemic Mice at Thermoneutrality

**Fig. 2.**
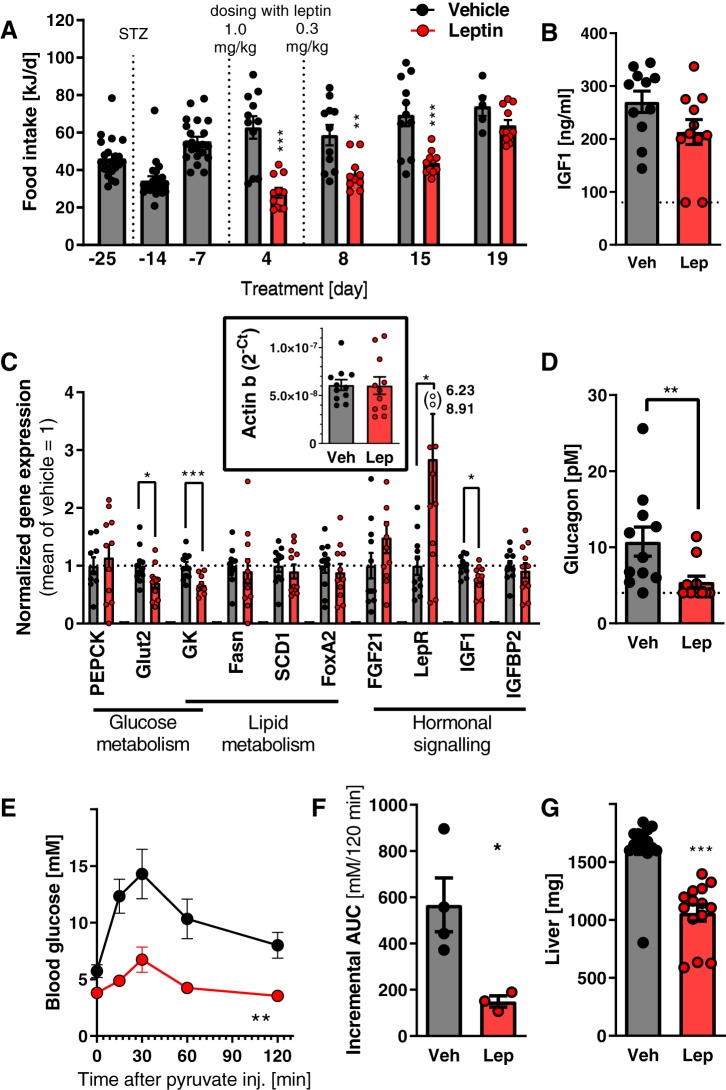
Metabolic effects of leptin on lean mice with streptozotocin-induced diabetes at thermoneutrality. Time course of changes in food intake after streptozotocin (STZ) treatment and during leptin (Lep) treatment (*A*). The food intake in vehicle-treated mice was increased by 50% at *day 19* versus *day −25* (*t* test; *P* < 0.005). Nonfasting plasma levels of IGF1 at time of dissection (*B*). Gene expression in liver (*C*) [the average of the vehicle (Veh)-treated group was set to 1; data normalized to β-actin as housekeeping gene; *top inset*]. For gene abbreviations, see Table 2. Nonfasting plasma levels of glucagon at the time of dissection (*D*). Blood glucose levels during pyruvate tolerance test performed at *day 7* in an independent cohort (*E*). Incremental area under glycemic curve during pyruvate test (i.e., basal blood glucose level is subtracted) (*F*). Liver weight (combined data from *studies 1* and *2*) (*G*). *Study 1*; *n* = 10–11 (*A*–*D* and *G*). *Study 2*; *n* = 3–4 (*E*–*G*). Statistics: Student’s *t* test (*A*–*D*, *F*, and *G*), repeated measures two-way ANOVA (*E*). AUC, area under the curve. *, **, and ***Significant effects of leptin treatment (*P* < 0.05, < 0.01, and < 0.001, respectively).

After the development of hyperglycemia, a 3-wk period of treatment with leptin was initiated. As seen in Fig. 1*A*, leptin treatment efficiently normalized blood glucose in these mice, principally as has been observed earlier (47). Furthermore, any further rise in glycated hemoglobin was prevented by the leptin treatment (Fig. 1, *B* and *C*). As expected, leptin also initially markedly decreased food intake (Fig. 2*A*). A reduction in body weight and fat mass was observed (Fig. 1, *D* and *F*). Food intake rose again toward the end of the experiment, demonstrating that the persistent effect of leptin on blood glucose was independent of the initially reduced food intake (compare Figs. 1*A* and 2*A* and see discussion).

To clarify the mode of action of leptin, we analyzed several suggested mediators. In our model, leptin did not decrease corticosterone levels (Fig. 1*J*), nor did it increase insulin (Fig. 1*G*) or IGF1 levels (Fig. 2*B*) (cf. Ref. 7), and IGF1 expression in the liver was even slightly decreased by leptin (Fig. 2*C*). Expression of IGF binding protein 2, also proposed to play a role in mediating the effects of leptin (20), was not affected by the treatment (Fig. 2*C*). In contrast, leptin treatment markedly suppressed plasma glucagon levels (Fig. 2*D*), in many mice to below the assay threshold. Thus, glucagon was the only parameter measured that showed a pattern that corresponded with the glucose-lowering effect of leptin.

Glucagon normally acts to elevate hepatic glucose production. We performed functional measurements of hepatic gluconeogenesis using a pyruvate tolerance test. In this experimental setting, leptin effectively blocked pyruvate-to-glucose conversion (Fig. 2, *E* and *F*), indicating suppressed rates of gluconeogenesis. Also, leptin treatment led to decreased weight of the liver (Fig. 2*G*), probably in part because of reduction of glycogen content as observed in similar experimental conditions (9).

Thus, leptin very efficiently ameliorated hyperglycemia in this new model of type 1 diabetes, probably through lowered hepatic glucose production resulting from lowered glucagon levels (Fig. 2*D*).

### UCP1 Is Not Involved in the Glucose-Lowering Effect of Leptin

Several mechanisms have been suggested to explain the glucose-lowering effect of leptin. One such mechanism is the uptake of glucose into certain tissues. BAT particularly may be suggested to mediate the glucose-lowering effect of leptin (7) based on its high capacity for glucose uptake (2).

**Fig. 3.**
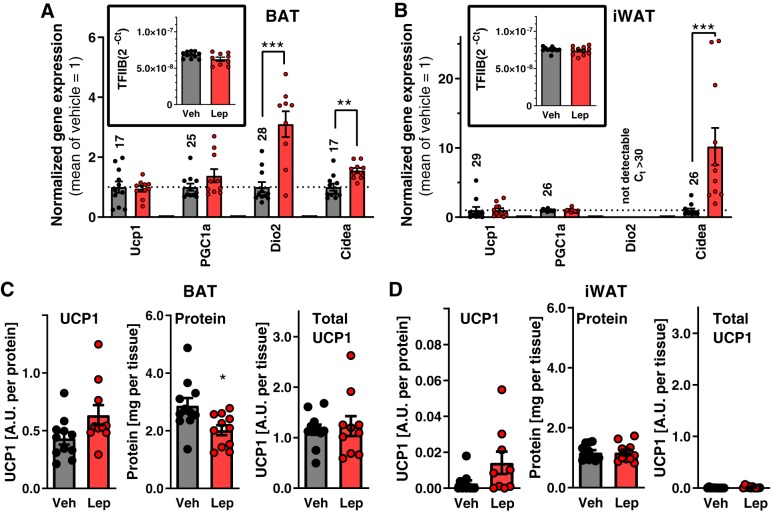
No effect of leptin (lep) on uncoupling protein-1 (UCP1) levels in brown adipose tissue (BAT) and inguinal white adipose tissue (WAT) in thermoneutral mice. Expression of genes associated with nonshivering thermogenesis and browning in BAT (*A*) and inguinal WAT (*B*) [data normalized to general transcription factor IIB (TFIIB) as housekeeping gene; *insets*]. The average of the vehicle (veh)-treated group was set to 1; average C_t_ values of vehicle-treated animals are displayed on top of each control column. Assessment of UCP1 protein content in BAT (*C*). UCP1 amount per protein unit [quantification of intensity of Western blot band normalized to a BAT standard on each blot after loading an equal amount (4 μg) of protein in each well (*left*), total protein in the tissue (*middle*), and total amount of UCP1 in the whole depot (total protein multiplied by UCP1 amount per protein; *right*)]. Tissue wet weights were 58.8 ± 2.0 mg in the vehicle-treated group and 22.3 ± 1.1 mg in the leptin-treated group. UCP1 protein content in inguinal WAT (iWAT) (*D*), as in *C*, but 9 μg protein was loaded; the same standard was used for normalization. Tissue wet weights were 103.0 ± 10.5 mg in the vehicle-treated group and 65.0 ± 5.2 mg in the leptin-treated group. *Study 1*; Bl/6J mice fed chow, *n* = 10–11. Statistics: Student’s *t* test; data sets that were not normally distributed [i.e., Ucp1 and deiodinase 2 (Dio2) in *A*, cell-death-inducing DNA fragmentation factor α subunit-like effector A (Cidea) in *B*, and UCP1/protein and total UCP1 in *D*] were evaluated by Mann–Whitney nonparametric test. PGC1a, peroxisome proliferator-activated receptor γ-coactivator 1 α. ** and ***Significant effects of leptin treatment (*P* < 0.01 and < 0.001, respectively).

Although leptin does not directly activate BAT thermogenesis in normal mice (12), it induces glucose uptake into BAT in type 1 diabetic animals (15). Chronic activation of BAT would normally lead to increased expression of UCP1. To establish whether there were such signs of chronic leptin-induced activation of the tissue, we examined the effect of the 3-wk leptin treatment of the type 1 diabetic mice on the expression of several genes associated with thermogenesis and mitochondrial biogenesis, both in interscapular BAT and in the inguinal white adipose tissue (iWAT) depot (Fig. 3, *A* and *B*). The iWAT depot is the white adipose tissue depot that is most prone to browning (i.e., the induction of brown-fat characteristics in classical white adipose tissue areas, resulting in “brite/beige” adipose tissue being formed) (5, 40).

In BAT, expression of UCP1, the critical component of nonshivering thermogenesis, remained unchanged, although the expression of the thermogenesis-related genes deiodinase 2 and cell-death-inducing DNA fragmentation factor α subunit-like effector A (Cidea) was enhanced by leptin treatment (Fig. 3*A*). Similarly, in inguinal WAT, UCP1 expression was not affected by leptin treatment (Fig. 3*B*). In this tissue, there was no detectable expression of deiodinase 2, whereas Cidea gene expression was markedly upregulated by leptin (Fig. 2*B*). It may be noted that all the thermogenic markers were much higher in BAT than in inguinal WAT (e.g., for UCP1 there was a difference of 12 C_t_ values, i.e., 4,000-fold difference in UCP1 mRNA levels).

As mRNA levels may not necessarily correspond to protein levels, the UCP1 protein content in both depots was assessed (Fig. 3, *C* and *D*). The amount of UCP1 protein per microgram protein tended to be increased in BAT after leptin treatment (Fig. 3*C*, *left*), but as the total protein amount in the tissue was slightly decreased (Fig. 3*C*, *middle*), there was no change in total UCP1 protein in the depot (Fig. 3*C*, *right*), implying that the total thermogenic capacity of the tissue was unchanged. In inguinal WAT, there was an almost undetectable amount of UCP1 (Fig. 3*D*), ~1/100 of the amount found in BAT. Thus, neither the level of UCP1 mRNA nor UCP1 protein was augmented in any of the studied depots, making it less likely that induction of UCP1 explains the glucose-lowering effect of leptin.

**Fig. 4.**
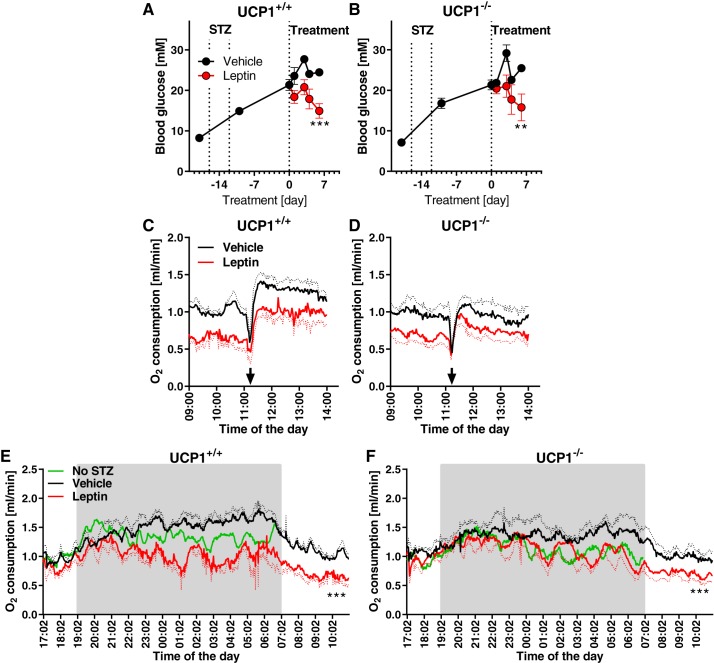
Glucose-lowering effect of leptin is uncoupling protein 1 (UCP1)-independent. Time course of nonfasted morning blood glucose in UCP1^+/+^ (*A*) and UCP1^−/−^ C57Bl6/N mice (*B*). Oxygen consumption in UCP1^+/+^ (*C*) and UCP1^−/−^ mice (*D*) after stimulation by 1 mg/kg β_3_-adrenergic agonist CL316,243 (dotted line represents SE; time of sc injection indicated by the arrow). Energy expenditure during the first hour postinjection is mainly a stress reaction in these nonanesthetised animals. The experiment was performed after 7 days of leptin treatment. At that time, vehicle-treated UCP1^+/+^ mice had 19.9 ± 0.3 g lean mass and 2.1 ± 0.2 g fat mass; leptin-treated UCP1^+/+^ mice had 19.7 ± 0.9 g lean mass and 1.6 ± 0.4 g fat mass; vehicle-treated UCP1^−/−^ mice had 18.0 ± 0.6 g lean mass and 1.9 ± 0.4 g fat mass; leptin-treated UCP1^−/−^ mice had 19.3 ± 0.8 g lean mass and 1.4 ± 0.4 g fat mass. Oxygen consumption in UCP1^+/+^ (*E*) and UCP1^−/−^ mice (*F*) measured for 18 h (shaded rectangle represents dark phase). The experiment was performed after 7 days of leptin treatment. The “no streptozotocin (STZ)” mice were from another but similar cohort. *Study 3*; C57 Bl/6N mice fed chow, *n* = 3–4. Statistics: two-way repeated measures ANOVA (vehicle ves. leptin). ***Significant effect of leptin treatment (*P* < 0.001).

However, although leptin did not increase the UCP1 protein amount, the UCP1 already present could mediate the glucose-lowering effect of leptin. To examine this, effects of leptin treatment on glucose levels were compared between diabetic UCP1^+/+^ and UCP1^−/−^ mice (of C57Bl/6N background). Leptin clearly decreased blood glucose to a similar extent in both UCP1^+/+^ and UCP1^−/−^ mice (Fig. 4, *A* and *B*). There is thus no requirement for UCP1 activity for the glucose-lowering effect of leptin (in agreement with Ref. 8, in which researchers studied UCP1^−/−^mice of C57Bl/6J background).

It may be argued that the physiological conditions in these mice (e.g., the hypoinsulinemia) are such that UCP1 is not functional. To examine this, we placed mice in metabolic chambers and activated the total UCP1-mediated nonshivering thermogenic capacity by injection of the selective β_3_-adrenergic agonist CL316,243. This resulted in a rapid and sustained increase in oxygen consumption in UCP1^+/+^ mice but not in UCP1^−/−^ mice (Fig. 4*C* vs. Fig. 4*D*), as expected (17). The magnitude of the rise in oxygen consumption in UCP1^+/+^ mice was comparable between vehicle- and leptin-treated mice [in agreement with results on leptin-treated euinsulinemic mice (12)]. Thus, in diabetic mice, leptin did not affect the thermogenic capacity of UCP1.

The basal level of oxygen consumption was strikingly different between vehicle- and leptin-treated diabetic mice, regardless of genotype (Fig. 4, *C* and *D*, before the injection). We therefore monitored oxygen consumption throughout the light and dark phases and compared the diabetic mice with a parallel group of mice not treated with streptozotocin (Fig. 4, *E* and *F*). Streptozotocin treatment increased oxygen consumption, as has been reported earlier (19). The mechanism behind this is unclear; it is clearly not mediated by UCP1 activation, but it agrees with the hyperphagia that develops in the streptozotocin-treated mice (Fig. 2*A*). Leptin treatment suppressed the increased oxygen consumption (Fig. 4, *E* and *F*); this effect could be due to the reduced food intake (Fig. 2*A*).

Taken together, these observations concerning UCP1 amounts and activity are therefore contrary to any hypothesis regarding a role for leptin-induced UCP1-mediated glucose combustion in BAT or brite/beige fat in the leptin-induced amelioration of hyperglycemia. However, UCP1-independent effects in these tissues cannot be excluded, as glucose uptake is not necessarily linked to UCP1-mediated glucose oxidation in these tissues (18, 35).

### Lack of Leptin Effect in High-Fat Diet-Fed Diabetic Mice at Thermoneutrality

**Fig. 5.**
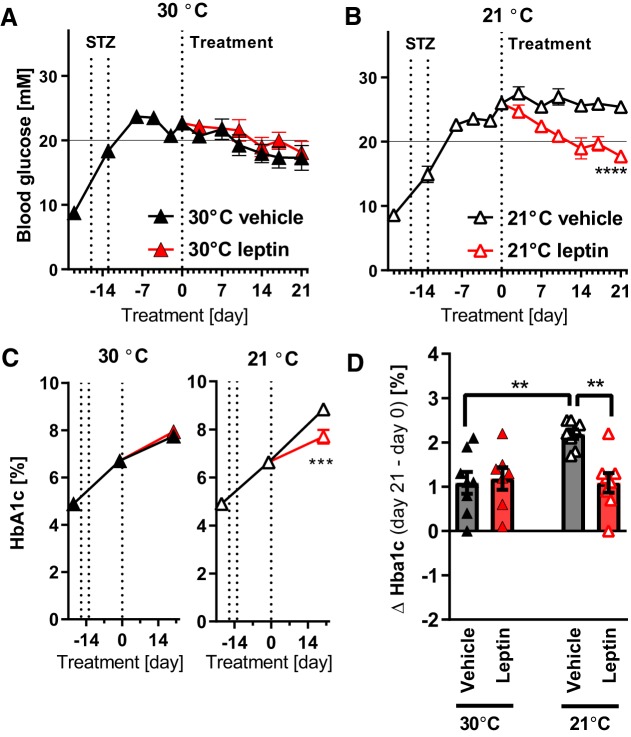
Glucose-lowering effect of leptin treatment in high-fat-diet-fed mice at 30°C and 21°C. *A* and *B*: time course of nonfasted morning blood glucose levels after streptozotocin (STZ) treatment and during leptin treatment in mice housed at thermoneutrality (*A*) or standard temperature (*B*). *C*: time course of glycated hemoglobin A1c (HbA1c) changes. *D*: changes in HbA1c during leptin treatment (between *days 0* and *21*). *Study 4*; Bl/6J mice fed 45% high-fat diet, *n* = 5–7. Statistics: one-way ANOVA with Tukey’s multiple comparison test (*D*); two-way repeated measures ANOVA alone (*A* and *B*) or together with Sidak’s multiple comparison test for comparison of individual time points (*C*). **, ***, and ****Significant effects of leptin treatment (*P* < 0.01, < 0.001, and < 0.0001, respectively).

Although thermoneutrality should be partially protective against fat loss, the streptozotocin-treated mice still lost fat and became hypoleptinemic (Fig. 1, *F* and *H*). As we sought a diabetes model with at least normal leptin levels, we examined a system in which a high-fat diet regime in mice at thermoneutrality preceded the streptozotocin treatment. In these mice, leptin treatment completely failed to affect blood glucose levels (Fig. 5*A*) or glycated hemoglobin (Fig. 5, *C* and *D*, 30°C).

Considering this unexpected absence of effect of leptin, we housed a parallel group of mice at standard animal house temperature. In comparison with the thermoneutral mice, mice housed at standard temperature with time developed somewhat higher blood glucose levels after streptozotocin treatment (Fig. 5*A* vs. Fig. 5*B*). Leptin treatment decreased glycemia in these mice to the levels in thermoneutral vehicle-treated mice (Fig. 5*A*). The changes in blood glucose were also reflected in the glycated hemoglobin levels: leptin prevented a sustained rise of HbA1c in mice at standard temperature, whereas at thermoneutrality, the rise of HbA1c was not as pronounced and leptin did not have any additional effect (Fig. 5, *C* and *D*). Thus, the inability to respond to leptin in the high-fat-fed mice kept at thermoneutrality may be said to be partly ameliorated in the mice at 21°C.

**Fig. 6.**
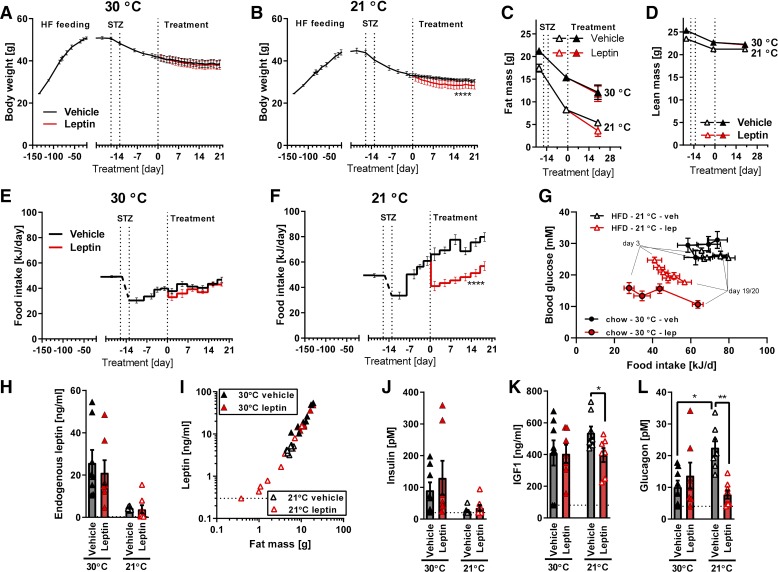
Effect of leptin (lep) treatment on metabolic parameters in high-fat (HF) diet (HFD)-fed mice at 21°C and 30°C. *A* and *B*: time course of body weight changes during high-fat-diet feeding, streptozotocin (STZ) treatment, and leptin treatment in mice housed at thermoneutrality (*A*) or standard temperature (*B*). *C*: time course of changes in body fat mass. *D*: time course of changes in lean body mass. Leptin data (red curves) fully coincide with vehicle (veh) data and are therefore not visible. *E* and *F*: time course of changes in food intake. *G*: correlation between food intake and blood glucose in high-fat diet-fed mice at 21°C (triangles; data corresponding to Figs. 5*B* and 6*F*) and chow-fed mice at 30°C (circles; data corresponding to Figs. 1*A* and 2*A*). *H*: nonfasting plasma levels of endogenous leptin. *I*: correlation between fat mass and endogenous leptin levels. *J*: nonfasting plasma levels of insulin. *K*: nonfasting plasma levels of IGF1. *L*: nonfasting plasma levels of glucagon. Data in *H*–*L* were all obtained at the time of dissection. *Study 4*; Bl/6J mice fed 45% high-fat diet, *n* = 5–7. Statistics: one-way ANOVA with Tukey’s multiple comparison test (*H*, *J*–*L*) or two-way repeated measures ANOVA alone (*A*, *B*, *E*, and *F*), or together with Sidak’s multiple comparison test for comparison of individual time points (*C* and *D*; no significant difference found). * and **Significant effects of leptin treatment (*P* < 0.05 and < 0.01, respectively).

We analyzed whether the less pronounced effects of leptin in the thermoneutral mice were related to the obesity of these mice and their corresponding leptin levels. At the time of streptozotocin treatment, the thermoneutral mice were already severely obese [approximately 50 g body weight and more than 20 g fat mass (Fig. 6, *A* and *C*, *day −16*)]. Streptozotocin treatment led to a loss of body weight (Fig. 6*A*), mainly because of a reduction in fat mass (Fig. 6, *C* and *D*, lines labeled as 30°C). However, even after 5 wk with a continuous decrease in body weight, the mice still retained more than 10 g body fat (Fig. 6*C*, 30°C), which was sufficient to sustain a level of 20 ng/mL endogenous leptin in the nonfasted state (Fig. 6*G*, *left* column).The observed body weight loss can be attributed to the persistent decrease in food intake after streptozotocin treatment (Fig. 6*E*). Leptin treatment did not reduce food intake further. Thus, the high levels of endogenous leptin in these mice are associated with resistance to both the anorexic and the glucose-lowering effects of injected leptin.

As expected, the mice at 21°C gained body weight more slowly (Fig. 6*B*), and their loss of body weight and fat mass after streptozotocin treatment was more pronounced, such that they retained only 5 g body fat at the end of the experiment (Fig. 6*C*, 21°C). Leptin led to an abruptly lowered food intake (Fig. 6*F*). This decrease in food intake indicated that in this respect, as well as in the glucose-lowering effect, the mice were not leptin resistant, in contrast to the mice at thermoneutrality (Fig. 6*E*). It may be suggested that this decrease in food intake could contribute to the observed decrease of blood glucose (Fig. 5*B*). However, as depicted in Fig. 6*G* (triangles), over the next weeks the decrease in blood glucose levels was enhanced, whereas the food intake was increased to successively reach preleptin-treatment levels. The lowered glucose level was therefore not understandable as being an effect of the decrease in food intake in itself.

Leptin levels were markedly lower than in the mice at thermoneutrality (Fig. 6*H*), in agreement with the decrease in body fat mass (Fig. 6*C*), and leptin treatment did not alter the relationship between body fat mass and endogenous leptin levels (Fig. 6*I*).

Leptin treatment had no effect on insulin levels (Fig. 6*J*) and only a small effect on IGF1 levels (Fig. 6*K*). However, also in these mice, changes in glucagon corresponded to glycemia: at thermoneutrality, glucagon was relatively low with no suppressing effect of leptin, whereas at standard temperature, in which glucagon levels were higher, leptin suppressed the glucagon levels at least down to the levels observed in mice at thermoneutrality (Fig. 6*L*), principally similarly to the effects of leptin on glucose levels.

Thus, also in these mice, there are clear parallelisms between leptin-sensitive food intake effects, glucagon-decreasing effects, and glucose-lowering effects.

### Leptin Effects in Mice Treated with Insulin Receptor Antagonist

From the above data, it may be implied that the efficiency of leptin treatment depends primarily on the endogenous leptin levels before treatment. To examine the importance of various leptin levels in an independent model of impaired insulin signaling, the insulin receptor antagonist S961 was used to remove insulin action in mice designed to have low or high leptin levels.

**Fig. 7.**
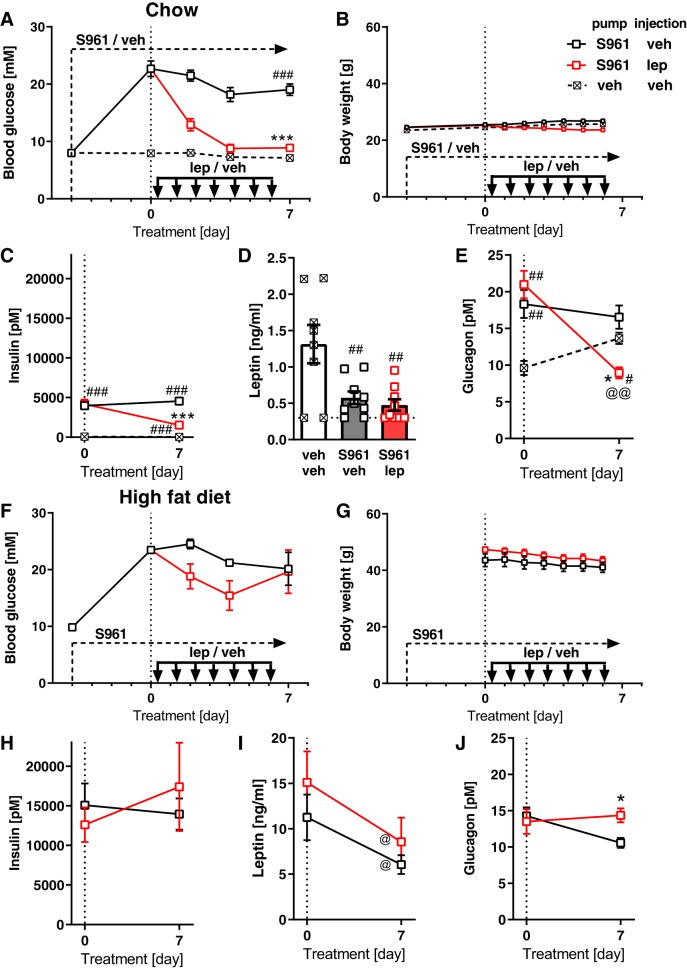
Effects of leptin treatment in chow-fed and high-fat-diet-fed mice with S961-induced diabetes. *A*: time course of nonfasting morning blood glucose levels during S961 and leptin (lep) treatments in chow-fed mice. *B*: time course of body weight changes in chow-fed mice. *C*: time course of nonfasting insulin levels in chow-fed mice. The insulin level in the vehicle (veh)-veh mice was ***≈***73 pM at *day 0* and 30 pM at *day 7*. *D*: endogenous leptin levels at *day 7* in chow-fed mice. *E*: time course of nonfasting glucagon levels in chow-fed mice. *F*: time course of nonfasting morning blood glucose levels during S961 and leptin treatment in high-fat-fed mice. *G*: time course of body weight changes in high-fat-fed mice. *H*–*J*: time course of nonfasted insulin (*H*), leptin (*I*), and glucagon (*J*) in high-fat-fed mice. *A*–*E*: *study 5*; Bl/6J mice fed chow, *n* = 6–10. *F*–*J*: *study 6*; Bl/6J mice fed 60% high-fat diet. Statistics: two-way repeated measures ANOVA with Tukey’s multiple comparison test (*A*, *B*, *F*, and *G*), or two-way ANOVA with Sidak’s multiple comparison test (*C*, *E*, and *H*–*J*), or one-way ANOVA with Tukey’s multiple comparison test (*D*). * and ***Significant difference in comparison with S961-vehicle group (*P* < 0.05 and < 0.001, respectively). #, ##, and ###Significant difference in comparison with vehicle-vehicle group (*P* < 0.05, 0.01, and 0.001, respectively). @ and @@Significant difference in comparison with *day 0* (*P* < 0.05 and < 0.01, respectively).

In chow-fed (lean) mice, S961 efficiently blocked insulin action, as the blood glucose levels reached values above 20 mM in just 4 days (Fig. 7*A*, until *day 0*). In this model, leptin treatment normalized glycemia in 4 days (Fig. 7*A*). There was no effect of this treatment on body weight (Fig. 7*B*). As expected, the hyperglycemia led to very high insulin levels compared with control mice (Fig. 7*C*, *day 0*); the levels decreased when the hyperglycemia was ameliorated (Fig. 7*C*). In spite of a stable body weight, blocking the insulin receptor resulted in a drop in leptin levels (compare nondiabetic vehicle (veh)-veh controls and S961-veh mice in Fig. 7*D*), as expected (43). Leptin treatment strongly decreased glucagon levels (Fig. 7*E*) in parallel with its effect on blood glucose.

Also, in high-fat-fed mice, S961 increased blood glucose levels to above 20 mM (Fig. 7*F*). In these mice, leptin treatment led only to a transient and nonsignificant reduction of blood glucose (Fig. 7*F*). Body weight remained high (Fig. 7*G*). Leptin treatment had no effect on the extremely high insulin levels (Fig. 7*H*). Endogenous leptin levels decreased during the leptin treatment (Fig. 7*I*) despite the fact that insulin was unchanged. Final plasma glucagon was even higher in the leptin-treated animals than in the vehicle-treated animals (Fig. 7*J*), indicating that leptin was unable to lower plasma glucagon, just as it was unable to lower blood glucose in these obese mice.

Thus, also in these pharmacologically diabetic mice, the ability of leptin treatment to lower glucose levels was governed by the initial leptin level: leptin decreased blood glucose when leptin levels were lower but lost this ability when initial leptin levels were higher.

## DISCUSSION

A possibility to use leptin in the treatment of patients with type 1 diabetes has been discussed. In the present study, the efficacy of leptin treatment to reduce blood glucose levels was investigated in model systems of type 1 diabetes. Whereas leptin was efficient in reducing blood glucose levels in mice with low endogenous leptin levels, it lost its efficacy in mice with high endogenous leptin levels, i.e., the mice became leptin resistant even in this respect. The glucose-reducing effect of leptin was not mediated via residual insulin, IGF1, IGF binding protein 2, corticosterone, food intake, or UCP1. However, leptin lowered glucagon levels, although this occurred only under hypoleptinemic conditions, an effect that thus correlated with the blood glucose-lowering effect of leptin. We thus suggest that the leptin effect is mostly mediated by suppression of the glucagon levels and, through this, by suppression of glucose release from the liver. Importantly, as a consequence of the leptin resistance demonstrated here, the therapeutic potential of leptin treatment in type 1 diabetes is likely limited.

### Efficiency of Leptin Treatment Is Attenuated by Higher Endogenous Leptin Levels

**Fig. 8.**
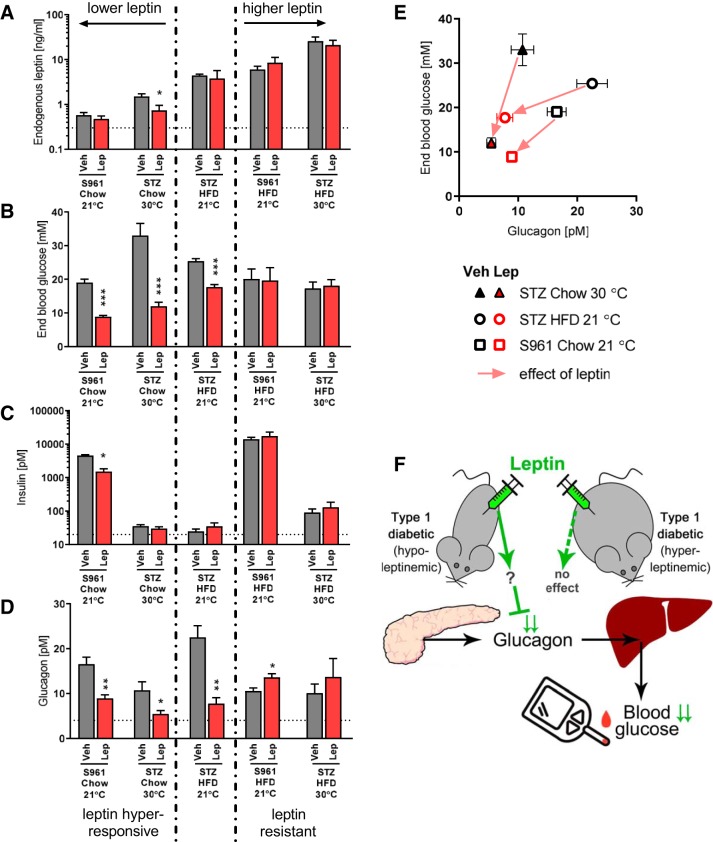
Endogenous leptin levels as determinants of exogenous leptin effects. Compilation of end point parameters from the previous figures; gray and red colors indicate final values in vehicle- and leptin-treated mice. *A*: final nonfasting plasma leptin levels in the individual indicated experiments; note the logarithmic scale. *B*: final blood glucose levels. *C*: final nonfasting plasma insulin levels; note the logarithmic scale. *D*: final nonfasting plasma glucagon levels. *E*: summary of effects of leptin treatment on glucagon and blood glucose in individual experiments (only data from experiments in which leptin achieved a significant effect on blood glucose levels). *F*: graphical summary. Glucose-lowering effects of leptin are dependent on endogenous leptin levels and are proposed to be mediated in part by suppression of glucagon secretion and glucose release from the liver. Statistics: Student’s *t* test for each pair of vehicle- and leptin-treated groups. *, **, and ***Significant difference in comparison to respective vehicle group (*P* < 0.05, < 0.01, and < 0.001, respectively). HFD, high-fat diet; STZ, streptozotocin.

For integral analysis, we have compiled the data from our different experimental series in Fig. 8. We ordered the different experiments according to the leptin levels in the mice in the absence of leptin treatment (Fig. 8*A*, gray columns; note the logarithmic scale). The chow-fed groups had very low leptin levels (<2 ng/mL leptin), the high-fat diet-fed animals housed at standard temperature had medium leptin levels (4–7 ng/mL), and high-fat diet-fed mice housed at thermoneutrality were hyperleptinemic (∼20 ng/mL).

The blood glucose levels in the different diabetes models did not reflect the endogenous leptin levels (Fig. 8*B*, gray columns). However, the glucose-lowering effect of leptin treatment was highly correlated with endogenous leptin levels (compare the red columns with the gray columns in Fig. 8*B* from left to right). As seen, leptin failed to lower the blood glucose levels in mice that had high endogenous leptin levels. It may not be considered surprising that the glucose-lowering effect of leptin shows leptin resistance, as do other effects of leptin (26). Indeed, we see a clear association between leptin effects on feeding and on glucose lowering: an absence of both of these effects is seen in Figs. 5*A* and 6*E*, while both these effects are observed in Figs. 1*A*, 2*A*, 5*B*, and 6*F*.

We have chosen here to alter the endogenous leptin levels through physiological means, i.e., mainly by alterations in the degree of obesity, resulting in the expected alterations in leptin levels (Figs. 1*I*, 6*H*, and 7). An alternative could have been to increase leptin, e.g., by a viral construct, but we have found the physiological regulation by high-fat diet and thermoneutrality more relevant. It has been discussed that the leptin resistance induced by high-fat diet could be due to a direct effect of the diet on the hypothalamus (proposed mechanisms reviewed in Ref. 10), rather than being secondary to the obesity-induced high leptin in itself. However, we see a glucose-lowering effect of leptin even in mice fed a high-fat diet at standard temperature (Fig. 8*B*, *middle* columns), in agreement with Ref. 26. Also, the mechanism behind the leptin desensitization is not of importance for the conclusions of the present study: under our conditions, leptin is only glucose lowering at low endogenous leptin levels.

### Mediation of the Leptin Effect on Glucose Levels

Several mechanisms have been suggested to explain the glucose-lowering effect of leptin in insulin-deficient animals. Here, we have examined whether some of these mechanisms can contribute to the observed effects in our models of type 1 diabetes.

#### Food intake.

As expected, in several of our experimental conditions, leptin not only decreases blood glucose but also decreases food intake. Therefore, at first glance, a reduced food intake could be suggested to explain the leptin-induced lowering of blood glucose observed here. However, although a reduction in food intake may be involved, analyzing the kinetics of these two leptin effects makes it clear that they are disparate. This is demonstrated by low glycemia persisting even after normalization of food intake in hypoleptinemic mice (Figs. 1*A* and 2*A*). Indeed, in Fig. 6*G*, we have compiled the data for food intake and blood glucose level from both Figs. 1*A* and 2*A* and Fig. 5*B* and 6*F*. It is clear that there is not a positive correlation between food intake and blood glucose level (if anything, the correlation is the reverse in the leptin-treated mice), and in both experiments, the food intake ultimately returns to preleptin-treatment levels, whereas the blood glucose levels remain lowered. The leptin-induced reduction in food intake thus cannot in itself explain the leptin effect.

#### Residual insulin.

There was always some residual insulin present in the streptozotocin-pretreated models (Fig. 8*C*, gray bars). Therefore, an augmentation of the residual insulin levels could have explained the glucose-lowering effect of leptin. However, no leptin-driven increases in residual insulin levels were observed (Fig. 8*C*), and residual insulin can thus in itself not explain the glucose-lowering effect of leptin. However, in itself, the presence of residual insulin may be important for the mediation of the leptin effect in our system (see below).

#### Hepatic gluconeogenesis.

The most important effect of leptin in type 1 diabetic rodents is thought to be suppression of hepatic glucose production (33, 38, 47), which may be caused by decreased availability of gluconeogenic substrates or by reduction of the capacity of the enzyme machinery. In accordance with this tenet, we observed that pyruvate-to-glucose conversion was strikingly suppressed by leptin (Fig. 2, *E* and *F*). Although this suppression could explain the lower glucose levels, the effect of leptin may be communicated to the liver through hormonal systems. Hepatic gluconeogenesis is stimulated both by glucocorticoids and by glucagon, and suppression of either of these hormones may therefore be responsible for the effect of leptin on blood glucose levels.

#### Glucocorticoids and the hypothalamus-pituitary-adrenal axis.

Suppression of glucocorticoids has been suggested to be involved in the acute glucose-lowering effects of leptin, especially in severe insulin deficiency (38). The levels of glucocorticoid in our model were relatively low (Fig. 1*J*) (i.e., not biased by stress), and leptin did not affect the corticosterone levels significantly. Thus, neither our study nor those of others studying models with less severe insulin deficiency (33) showed a connection between corticosteroid levels and the glucose-lowering effect of leptin, making the involvement of the hypothalamus-pituitary-adrenal axis in the mediation of the leptin effect in our model less likely.

#### Glucagon.

Besides glucocorticoids, glucagon is the major regulator of hepatic glucose production. Leptin has been shown to lower glucagon secretion both directly (44) and via sympathetic nervous system activity (31), making suppression of glucagon levels an attractive candidate for mediating the effects of leptin.

Leptin reduced circulating glucagon in all the models that were leptin sensitive with respect to a glucose-lowering effect (Fig. 8*D*). Out of all the parameters measured, changes in glucagon levels corresponded most closely with effects on blood glucose, although the correlation differed somewhat between the different experimental paradigms (Fig. 8*E*). Thus, in these mouse models, the endogenous leptin level appears to determine the magnitude of the effects of exogenous leptin, and the leptin effect may be mediated by glucagon. It could therefore be interesting to follow up the present studies with studies in which mice are kept hyperglucagonemic.

The nature of the pathway that links leptin to decreased glucagon was not established in our models, but mechanisms have been suggested (reviewed in Ref. 7).

Traditionally, glucagon is thought to affect gluconeogenesis mainly by induction of gluconeogenic gene expression. However, in our experiments, the phosphoenolpyruvate carboxykinase mRNA level was not decreased by leptin treatment in spite of clear changes in glucagon levels and rates of gluconeogenesis. Glucagon may also affect gluconeogenesis independently of regulation of gene expression, for instance by stimulation of amino acid uptake and thus increased availability of gluconeogenic substrates (25). Suppression of these mechanisms may play a role in our models as well.

Although a decrease in glucagon level does not always induce amelioration of hyperglycemia (1, 16, 29, 34) and although glucose-lowering effects have been reported to precede a decrease in glucagon levels in models of type I diabetes with total insulin deficiency (38), reduction of glucagon levels is the candidate best associated with the mediation of the leptin effects in our models. It would also seem that the fact that there is some residual insulin in our insulin-deficient animals allows the changes in glucagon level to be manifest in blood glucose (in agreement with Refs. 3 and 4).

Our interpretation of the data presented here is summarized in Fig. 8*F*, which highlights glucagon as the most likely mediator of the glucose-lowering effect of leptin.

### Human Perspective

A major reason for the investigation of the mechanism of action of leptin presented here is the potential use of leptin therapeutically. Leptin corrects insulin resistance and hyperglycemia in patients with hypoleptinemia and lipodystrophy (36, 39) and in patients presenting with a combination of lipodystrophy and type 1 diabetes (37). However, the effects have usually been less pronounced in patients with lipodystrophy but without hypoleptinemia (3a).

Patients with obesity (28) and type 2 diabetes (30, 32) are usually hyperleptinemic and thus resistant to the effects of leptin therapy. In patients with newly diagnosed type 1 diabetes, the endogenous leptin levels are generally decreased (42), and they are also very low in patients with diabetic ketoacidosis but rise again after initiation of insulin therapy, possibly because of a direct effect of insulin on leptin release (43). Thus, in contrast to traditional rodent models of type 1 diabetes, most patients with type 1 diabetes are not hypoleptinemic, and their sensitivity to leptin is therefore reduced.

We therefore consider that the insulin-deficient murine models presented here with sustained endogenous leptin levels mimic the human type 1 diabetes phenotype better than previously described animal models. These results are in accordance with a recent clinical study investigating leptin therapy in patients with type 1 diabetes with normal endogenous leptin levels. This study did not report any improvement in glycated hemoglobin levels by leptin treatment (45). Thus, experimental and clinical studies concur in the conclusion that, although leptin may possess an insulin-independent glucose-lowering effect, this effect has already become manifest in patients with insulin-treated type 1 diabetes, and additional beneficial effects on blood glucose levels of leptin treatment are therefore not to be expected.

## GRANTS

The work was supported by a STAR postdoc project from Novo Nordisk and by grants from the Swedish Research Council.

## DISCLOSURES

J. Nedergaard and B. Cannon are consultants of Atrogi. B. Andersen, K. Raun, G. Rakipovski, J. F. Paulsson, K. W. Conde-Frieboes, and J. J. Fels are employees of Global Drug Discovery, Novo Nordisk A/S.

## AUTHOR CONTRIBUTIONS

P.Z., J.F.P., K.W.C.-F., K.R., B.A., B.C., and J.N. conceived and designed research; P.Z., G.R., M.H.B., O.B., and J.J.F. performed experiments; P.Z., G.R., M.H.B., and O.B. analyzed data; P.Z. and J.N. interpreted results of experiments; P.Z. and J.N. prepared figures; P.Z. and J.N. drafted manuscript; P.Z., G.R., M.H.B., O.B., J.F.P., K.W.C.-F., K.R., B.A., B.C., and J.N. edited and revised manuscript; P.Z., G.R., M.H.B., O.B., J.F.P., K.W.C.-F., J.J.F., K.R., B.A., B.C., and J.N. approved final version of manuscript.
